# Oviposition behavior is not affected by ultraviolet light in a butterfly with sexually‐dimorphic expression of a UV‐sensitive opsin

**DOI:** 10.1002/ece3.10243

**Published:** 2023-07-04

**Authors:** Jose Borrero, Daniel Shane Wright, Caroline Nicole Bacquet, Richard M. Merrill

**Affiliations:** ^1^ Division of Evolutionary Biology LMU Munich Munich Germany; ^2^ Universidad Regional Amazónica de Ikiam Tena Ecuador

**Keywords:** behavior, *Heliconius*, oviposition, sexual dimorphism, UV, vision

## Abstract

Animal vision is important for mediating multiple complex behaviors. In *Heliconius* butterflies, vision guides fundamental behaviors such as oviposition, foraging, and mate choice. Color vision in *Heliconius* involves ultraviolet (UV), blue and long‐wavelength‐sensitive photoreceptors (opsins). Additionally, *Heliconius* possess a duplicated UV opsin, and its expression varies widely within the genus. In *Heliconius erato*, opsin expression is sexually dimorphic; only females express both UV‐sensitive opsins, enabling UV wavelength discrimination. However, the selective pressures responsible for sex‐specific differences in opsin expression and visual perception remain unresolved. Female *Heliconius* invest heavily in finding suitable hostplants for oviposition, a behavior heavily dependent on visual cues. Here, we tested the hypothesis that UV vision is important for oviposition in *H. erato* and *Heliconius himera* females by manipulating the availability of UV in behavioral experiments under natural conditions. Our results indicate that UV does not influence the number of oviposition attempts or eggs laid, and the hostplant, *Passiflora punctata*, does not reflect UV wavelengths. Models of *H. erato* female vision suggest only minimal stimulation of the UV opsins. Overall, these findings suggest that UV wavelengths do not directly affect the ability of *Heliconius* females to find suitable oviposition sites. Alternatively, UV discrimination could be used in the context of foraging or mate choice, but this remains to be tested.

## Graphical abstract

Our study focuses on the evolution of sexual dimorphism of UV vision in Heliconius butterflies. Under naturalistic light conditions, we experimentally altered UV‐light availability using light filters to test the hypothesis that, in *H. erato*, UV vision is important for oviposition, a female‐specific behavior. Our experiments suggest that UV perception in *H. erato* females is not used during oviposition.

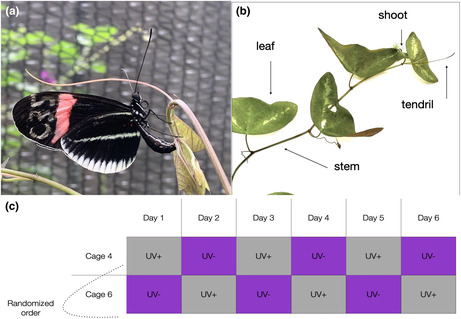

## Resumen

La visión animal cumple una función crucial guiando comportamientos complejos. Para las mariposas *Heliconius*, la visión juega un papel principal en comportamientos como la búsqueda de alimento, la elección de pareja y la ovoposición. La visión a color en *Heliconius* está compuesta por una combinación de fotoreceptores (opsinas) sensibles a rayos ultravioleta (UV), azul y ondas de longitud larga (verde‐rojo). Adicionalmente, estas mariposas posen una segunda opsina sensible a rayos UV, generada por una duplicación. La expresión de estas dos opsinas UV varia ampliamente dentro del genero de *Heliconius.* En la especie *Heliconius erato*, la expresión de estas dos opsinas presenta un dimorfismo sexual donde únicamente las hembras expresan las dos opsinas sensibles a UV, lo que les permite la discriminación de ondas en el rango ultravioleta. Sin embargo, no se han estudiado las presiones ecológicas que han llevado a estas diferencias en la percepción visual entre ambos sexos. Las hembras de *Heliconius* invierten mucho tiempo buscando plantas hospederas para poner sus huevos y este comportamiento depende en gran medida de señales visuales. En este estudio, manipulamos la disponibilidad de rayos UV en condiciones naturales de luz, para evaluar si el comportamiento de ovoposición es guiado por la visión en UV en *H. erato* y su especie hermana *Heliconius himera*. Descubrimos que la presencia de rayos ultravioleta no influye en el número de intentos de ovoposición ni en la cantidad de huevos puestos. Además, la planta hospedera *Passiflora puntata*, presenta una escasa reflexión en las longitudes de onda UV. Así mismo, nuestros modelos de la visión de *H. erato* hembras, predicen solamente una estimulación mínima de las opsinas UV. En resumen, nuestros resultados sugieren que las ondas ultravioletas no afectan directamente la capacidad de las hembras *Heliconius* para encontrar sitios de oviposición adecuados. Alternativamente, la discriminación de ondas UV podría estar siendo utilizada en el contexto de la búsqueda de alimento o la elección de pareja, pero esta hipótesis sigue por ser evaluada.

## 1 INTRODUCTION

Animal color vision mediates a multitude of complex behaviors. In general, color vision is achieved via wavelength discrimination (independent of intensity), where the inputs of two different photoreceptors (i.e., opsins, which differ in spectral sensitivity) are compared (Kelber, 1999; Kelber & Pfaff, 1999). In most insects, color vision is based on three photoreceptor classes, encoded by opsin genes, sensitive to ultraviolet (UV), blue (B), and long wavelengths (LW; Briscoe & Chittka, 2001; Kelber, 2006). The visual systems of butterflies are highly diverse with differing numbers of photoreceptor classes and sensitivities among families, genera, species, and even between sexes (Briscoe, 2008; McCulloch et al., 2017; Van Der Kooi et al., 2021). Visual system diversification in Lepidoptera has occurred through independent opsin duplications and is attributed to changes in light availability and habitat use (Sondhi et al., 2021).

Vision plays a crucial role in *Heliconius* behavior and guides fundamental processes such as foraging (Toure et al., 2020), interspecific and intraspecific communication (Estrada & Jiggins, 2008; Jiggins et al., 2001; Merrill et al., 2014) and hostplant selection (Gilbert, 1982). However, few studies have investigated the role of *Heliconius* visual systems from an ecological context (Dell'Aglio et al., 2018; Finkbeiner et al., 2017). Relative to other butterflies, *Heliconius* have some of the largest brains and invest heavily in the visual neuropile, suggesting selection for well‐developed vision (Montgomery et al., 2016). In addition to the UV (*UVRh1*), blue (*BRh*), and long‐wavelength (*LWRh*) sensitive opsins found in most insects, some species in the *Heliconius* genus possess a duplicated UV opsin (*UVRh2*; Briscoe et al., 2010). Additionally, some *LWRh*‐expressing cells possess lateral filtering pigments that shift the spectral sensitivity toward red, enabling *Heliconius* to discriminate wavelengths in the long‐wavelength range (McCulloch et al., 2022; Zaccardi et al., 2006).

Across several species of the *Heliconius* genus, opsin expression is variable. Some species, such as *Heliconius melpomene*, have independently lost expression of one of the two UV opsins, with documented pseudogenization events (McCulloch et al., 2017). *Heliconius erato* is sexually dimorphic—males only express *UVRh2* whereas females express *UVRh1* and *UVRh2* (Figure S1; McCulloch et al., 2016, 2017). It is possible that in *H. erato*, *UVRh1* is located in the W‐chromosome such as in *H. charithonia* (Chakraborty et al., 2022). A recent study investigated whether *H. erato* was capable of discriminating UV wavelengths: Finkbeiner and Briscoe (2021) tested *H. erato* females (which expresses *UVRh1* and *UVRh2*), *H. erato* males (expresses only *UVRh2*) and *H. melpomene* (both sexes express only *UVRh1*) in a laboratory experiment and found that only *H. erato* females can discriminate between UV wavelengths. However, the ecological pressures that have driven these species‐ and sex‐specific differences in visual perception remain unresolved.

While changes in opsin expression patterns within the *Heliconius* genus are well‐documented (Catalán et al., 2019; McCulloch et al., 2016, 2017, 2022), the adaptive function of these changes in gene expression remains unclear. Understanding the selective pressures for UV discrimination in *H. erato* females may give us insight as to why expression of this gene greatly varies between and within *Heliconius* species. In contrast to males, female *Heliconius* spend most of their time searching for host plants for oviposition, which involves careful visual inspection (Benson, 1978; Brown Jr., 1981). Females also avoid laying on hostplants where other eggs or larvae are present and *Passiflora* have evolved extra‐floral nectaries that reassemble yellow eggs to discourage ovipositing females (Williams & Gilbert, 1981). *H. erato* females use leaf shape as an oviposition cue and can learn new shapes, driving the evolution of leaf shape plasticity in *Passiflora* (Dell'Aglio et al., 2016). The importance of choosing suitable *Passiflora* vines suggests that this behavior is under strong natural selection (Jiggins, 2017), and there is evidence that *Heliconius* female vision is fundamental for finding suitable oviposition sites. Therefore, UV wavelength discrimination in *H. erato* females may be an adaptation to facilitate hostplant recognition, but the role of UV vision in this regard has not been tested.

If the microhabitats used by different *Heliconius* species vary in their light properties, particularly in the UV range, natural selection may drive changes in the expression patterns of the duplicated UV opsins. The closely related species *Heliconius erato cyrbia* and *Heliconius himera* present an opportunity to test this hypothesis. *H. erato cyrbia* inhabits low‐altitude secondary rainforest and *H. himera* is endemic to high altitude open dry forests in the western slopes of the Andes in southern Ecuador and Peru (Jiggins et al., 1996). These habitats may represent highly contrasting light environments; open forests receive direct sunlight, whereas dense shady forests are characterized by a “yellow‐green” light spectrum due to reflection from the leaves (Endler, 1993). UV radiation also increases with altitude (Blumthaler et al., 1997). Prior work has suggested environment‐specific adaptations between these species: there is neuroanatomical divergence between *H. erato cyrbia* and *H. himera*, with the former showing higher investment in sensory regions of the brain important for visual processing, such as color vision (Montgomery & Merrill, 2017). This may be due to *H. erato cyrbia* living in a more complex and challenging visual environment.

Here, we test whether UV vision is used for oviposition in *H. erato* females and its closely related species *Heliconius himera* by manipulating UV wavelength availability. We address the following questions: (1) Does UV light affect oviposition behavior in *H. erato cyrbia and H. himera*? Given that UV is found under natural sunlight conditions, we expect that reducing UV availability will lead to a reduction in the number of eggs laid. (2) Does this behavior differ between species? Compared with *H. himera*, *H. erato* has a larger investment in the visual system (Montgomery & Merrill, 2017), we predict a stronger effect in this species. (3) How are UV cues in the hostplant perceived by females? Given that *Heliconius* females preferentially lay eggs on young nutritious shoots (Benson et al., 1975; Jiggins, 2017), we predict that UV reflectance will be highest for this part of the hostplant. Finally, we use visual modeling to quantify the stimulation of the *H. erato* visual system by its hostplant *P. punctata*.

## 2 MATERIALS AND METHODS

### 2.1 Butterfly rearing and maintenance

Wild *Heliconius erat*o cyrbia were caught in forests near Balsas (3°51′26.0″S 79°34′05.3″W) and *H. himera* near Vilcabamba (4°15′57.3″S 79°13′41.4″W), in Southern Ecuador. Wild individuals were used to establish stocks at the Universidad Regional Amazónica Ikiam, Ecuador. The insectaries at IKIAM are in a clearing within a secondary forested area, reflecting the natural habitat of *H. erato* which are found at the forest edge (Jiggins et al., 1996). Butterfly stocks were kept in outdoor insectaries, in 2 × 2 × 2.3 m cages, fed a 20% sugar solution and had access to pollen from *Lantana* sp. and *Psiguria* sp. flowers. Eggs were collected from hostplants *P. punctata* (Jiggins et al., 1997), and the larvae were individually reared in pots on fresh leaves from *P. punctata*. For behavioral trials, we tested a total of 26 *H. erato cyrbia* and *10 H. himera* females (less *H. himera* were tested due to low butterfly stocks).

### 2.2 Experimental design

Experiments were conducted under natural sunlight conditions. To manipulate UV in the light environment, two experimental cages (100 × 200 × 235 cm; Figure S2) were fitted with either clear UV‐blocking (transmission 400–750 nm; LEE #226) or UV‐transmitting filter sheets (transmission 300–750 nm; LEE #130). The filters were attached to the top, left, and outward‐facing sides of the cage to filter the morning sunlight coming from the southeast (Figure S2). These light filters are frequently used in behavioral experiments to mimic natural light conditions or to exclude certain wavelengths altogether (Greenwood et al., 2002; Hiermes et al., 2021; Honkavaara et al., 2008; Veen et al., 2017; Wright et al., 2017).

The filters were attached to the top, left and outward‐facing sides of the cage to filter the morning sunlight coming from the southeast (Figure S2). We only fitted filters to these sides of the cages in order to prevent the cages from overheating. Because our experimental cages are in an outdoor insectary with plastic roofing made from polyethylene which blocks UV (Diaz & Fereres, 2007), there is effectively no source of UV light from the inside‐ and right‐facing sides of the cages (Figure S4). We conducted the experiments in the morning, meaning that most of the incoming sunlight came from the east (downwelling, side welling left, and side welling out directions of the experimental cages). We took multiple irradiance measurements before, during and after the behavioral experiments to ensure that wavelengths in the UV range were being filtered out (see Figures S3 and S4). There was effectively no UV light from the inside‐ and right‐facing sides of the cages (Figure S4). The UV‐blocking filters successfully reduced the amount of UV wavelengths (300–400 nm) present in the experimental cages (Figure S4). In contrast, UV wavelengths were present in the control UV+ treatment (i.e., with clear filters). The rest of the light spectrum—between 400 and 700 nm—remained unchanged between treatments (Figure S3).

**FIGURE 1:**
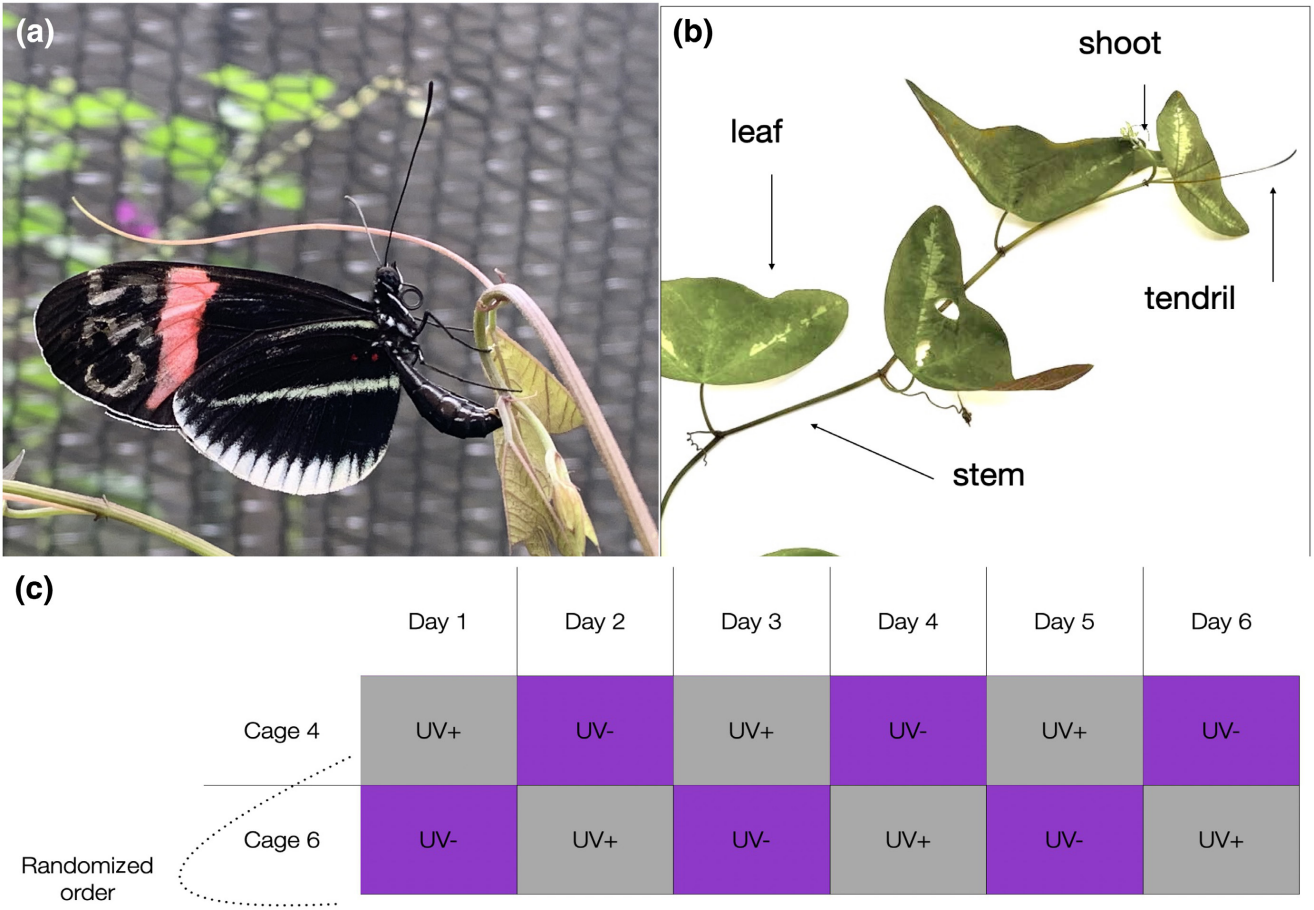
Overview of the experimental setup. (a) *Heliconius erato cyrbia* female attempting to lay an egg on a *Passiflora puctata* shoot. (b) Parts of hostplant *P. punctata*, where *H. erato* lay eggs. (c) Protocol of behavioral experiment.

The experimental assay lasted 6 days, during which, a group of females (1–6 individuals) was introduced into each experimental cage. As the female butterflies were chosen randomly from the stock cages, some individuals (10 *H. erato cyrbia* and 1 *H. himera*) were tested twice (thus, these individuals were tested in 12 trials instead of 6). Individuals were introduced to the experimental cages 24 h prior to the experiment to acclimate and were confirmed to have laid eggs on a hostplant in the experimental cage overnight. On the first day of the experiment, the two cages were randomly assigned a light treatment (UV+ or UV−), thereby controlling for changes in natural sunlight during the assay by testing both treatments in parallel. The following days, each group was tested with the opposite treatment, alternating light treatments three times (UV+/UV−; Figure 1c).

Before each trial, the filters were fitted to the experimental cages and a *P. puctata* hostplant was placed at the center of the cage. For each trial, the butterflies were observed for 2 h between 8:30 and 12:00. During this time of the day, *Heliconius are active*, and females lay eggs (Jiggins, 2017). Based on the placement of the filters in the experimental cages (Figure S2), we conducted the experiments in the morning when the majority of sunlight comes from the east. Females could freely fly inside the cage, feed on artificial feeders and a pollen plant, and lay eggs. For each individual, we recorded the number of oviposition attempts, number of eggs laid, and the hostplant “part” (shoot, leaf, tendril, or stem) where the egg was laid (Figure 1b). Oviposition attempts were scored as each time a butterfly landed on a hostplant and moved its abdomen with the ovipositor toward the plant, and each movement of the abdomen toward the plant was counted as an individual oviposition attempt. The sum of the three trials with the same treatment was combined for analysis.

### 2.3 Light measurements

Light measurements were taken using a Flame Miniature Spectrometer (Ocean Optics Inc.) connected to a UV–VIS optical fiber (P400‐2‐UV–Vis) with a cosine corrector (Ocean Optics CC‐3‐UV). In the morning (8:00–12:00), downwelling and side welling irradiance (in μmol/(m^2^*s)) was measured in the two experimental cages under the different light treatments (UV+/UV−). For all measurements, the weather conditions were categorized as sunny (<50% cloud coverage (cc.)), cloudy (>50% cc.), and overcast (100% cc.).

### 2.4 Reflectance spectrometry and visual modeling

Reflectance measurements of the hostplant *P. punctata* were taken using a Flame Miniature Spectrometer connected to a PX‐2 xenon light source (spectral range 220–750 nm) and a UV/Vis reflection probe (Ocean Optics Inc.). All reflectance measurements were standardized with a white reflectance standard (Ocean Optics WS‐1). For reflectance measurements, the illuminating and reflection probe was placed at a 45° angle at 1 mm from the plant tissue using a probe holder that was constructed in‐house. We recorded three measurements per plant (shoot, stem, leaf; integration time: 2500 ms per scan). Irradiance and reflectance measurements were processed and visualized using the pavo 2.2.0 package (Maia et al., 2019). For each plant tissue, three biological replicates were measured across five individual plants (45 measurements per plant part).

The visual perception of the hostplant was modeled with previously published *H. erato* visual system data (McCulloch et al., 2016, 2022) using the pavo 2.2.0 package (Maia et al., 2019). For the visual model, we used the following photoreceptor sensitivities of *H. erato* females: *UVRh1* λ_max_ 355 nm, *UVRh2* λ_max_ 390 nm, *BRh* λ_max_ 470 nm, *LWRh‐green* λ_max_ 555 nm, and a fifth photoreceptor class *LWRh‐red* λ_max_ 590 nm that occurs through expression of a red filtering pigment in combination with the green rhodopsin (McCulloch et al., 2016, 2022). We then calculated the photoreceptor quantum catch, which estimates the light captured by the visual system (Kelber et al., 2003) under each experimental light environment condition (UV+/UV−) against a green foliage background (Maia et al., 2013). The quantum catches were calculated as:

$$Q_{\mathrm{ci}}=\int_{300\mathrm{nm}}^{700\mathrm{nm}}I\left(\lambda \right)S\left(\lambda \right)R\left(\lambda \right)$$

where *I*(*λ*) is the irradiance measured in the experimental light conditions, *S*(*λ*) is the reflectance spectrum of the stimulus and *R*(*λ*) is the photoreceptor sensitivity based on the equations of (Govardovskii et al., 2000; Hart & Vorobyev, 2005).

### 2.5 Statistical analysis

Statistical analyses were conducted in R (R Core Team, 2023), and plots were created with the *ggplot2* package (Wickham, 2011; see Dryad repository for script in R Markdown). We fitted generalized linear‐mixed models (GLMM) with the *glmer* function in the lme4 package (Bates et al., 2015) to test whether oviposition behavior was affected by the presence or absence of UV and tested how the number of oviposition attempts and/or eggs laid was influenced by the fixed effects (and their interactions): (i) treatment (UV+/UV−), (ii) weather (<50% cloud coverage/>50% cc./100% cc.) and (iii) species (erato/himera). Where GLMMs with Poisson distribution were overdispersed, we fitted negative binomial models with the *glmer.nb* function in the lme4 package. To avoid pseudoreplication (individuals were tested multiple times), individual id was included as a random factor. The random effect structure of the full models was selected based on Akaike comparisons, choosing the model with the lowest AIC value (ΔAIC >4; Burnham & Anderson, 2004; Sakamoto et al., 1986). Stepwise model reduction of the fixed effects based on statistical significance (Crawley, 2002) was then conducted using likelihood ratio tests (LRT) via the *drop1* function to identify the minimum adequate statistical models. To estimate the parameters of significant fixed effects, we used parametric bootstrapping (nsim = 1000, pbkrtest package (Halekoh & Højsgaard, 2014)). For fixed effects with more than two categories (e.g., weather), we conducted pairwise comparisons using post hoc Tukey corrections with the emmeans package (Lenth et al., 2019).

## 3 RESULTS

### 3.1 UV does not affect oviposition behavior

**FIGURE 2:**
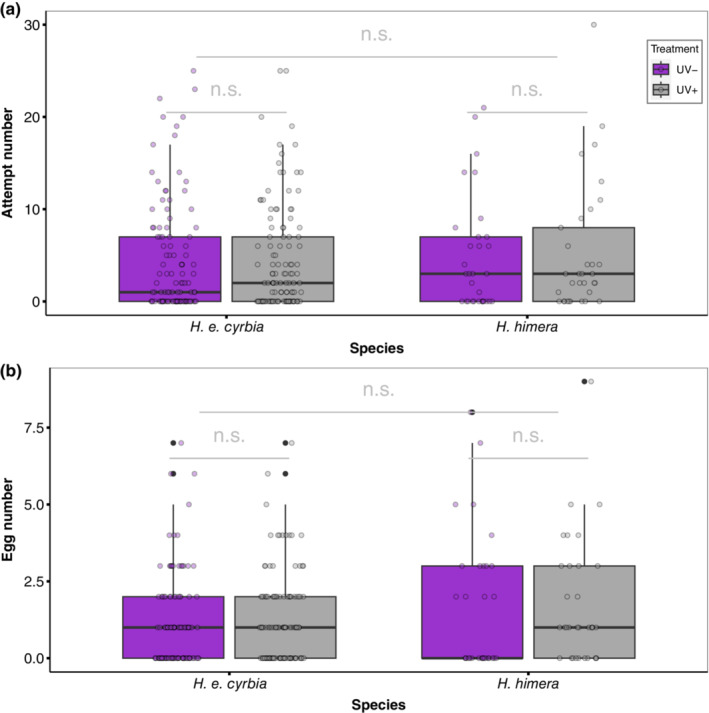
UV manipulation did not affect the (a) number of oviposition attempts or (b) the number of eggs laid on the hostplant. Gray boxes represent the number of attempts/eggs in the control treatment (UV+) and purple boxes represent the number of attempts/eggs in the UV‐light environment. Error bars represent ±1 standard error.

The availability of UV wavelengths did not significantly affect the number of oviposition attempts (LRT = 0.8055, df = 1, *p* = .39; “emmeans” contrast assessment: β ± SE = 0.152 ± 0.17; 95% CI = −0.181, 0.486; Figure 2a). Similarly, there were no species differences in the number of oviposition attempts (LRT = 0.1459, df = 1, *p* = .72; “emmeans” contrast assessment: β ± SE = −0.102 ± 0.266; 95% CI = −0.624, 0.419). Neither the UV treatment (LRT = 1.6258, df = 1, *p* = .20; “emmeans” contrast assessment: β ± SE = 0.161 ± 0.127, 95% CI = −0.0872, 0.41; Figure 2b) nor species identity (LRT = 1.0624, df = 1, *p* = .31; “emmeans” contrast assessment: β ± SE = −0.24 ± 0.225, 95% CI = −0.681, 0.202) had a significant effect on the number of eggs laid. When compared to the reported number of eggs laid in prior studies of *H. erato* (Hausmann et al., 2023), the number of eggs laid within a 2‐h window did not differ from the number of eggs laid in this behavioral experiment (Mann–Whitney *U*‐test, *W* = 15,242, *p* = .8389).

**FIGURE 3:**
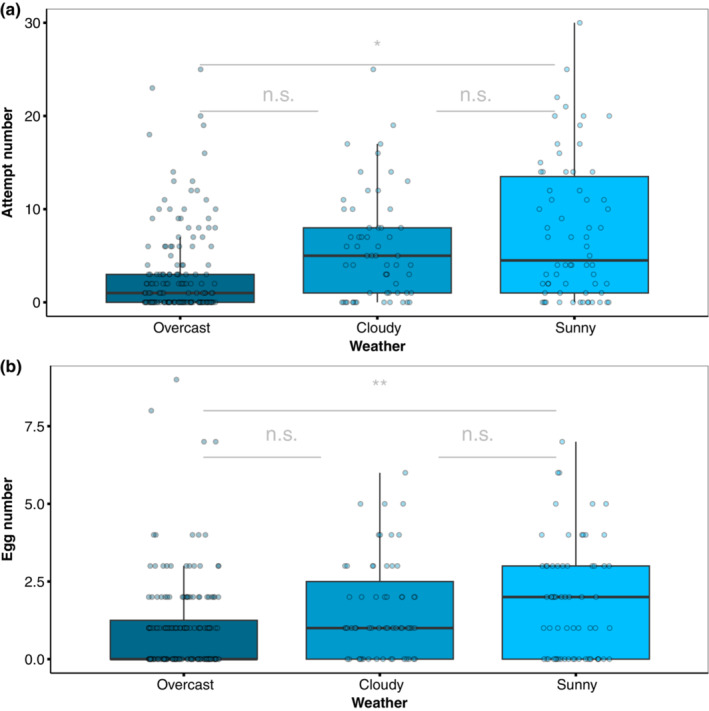
(a) Number of oviposition attempts per weather category and (b) number of eggs laid per weather category.

The number of oviposition attempts significantly differed by weather (LRT = 21.764, df = 2, *p* = .001; Figure 3a). Post hoc pairwise comparisons indicated that females had fewer attempts on days with full cloud coverage than on sunny days (*Z* = −2.837, *p* = .0127; “emmeans” contrast assessment: β ± SE = −0.945 ± 0.21, 95% CI = −1.370, −0.52). Weather also had a significant effect on the number of laid eggs (LRT = 11.641, df = 2, *p* = .004); more eggs were laid on sunny days (<50% cc.) than on overcast (100% cc.) days (*Z* = −2.446, *p* = .038; “emmeans” contrast assessment: β ± SE = −0.499 ± 0.162, 95% CI = −0.817, −0.180; Figure 3b).

### 3.2 Females prefer to lay eggs on shoots

**FIGURE 4:**
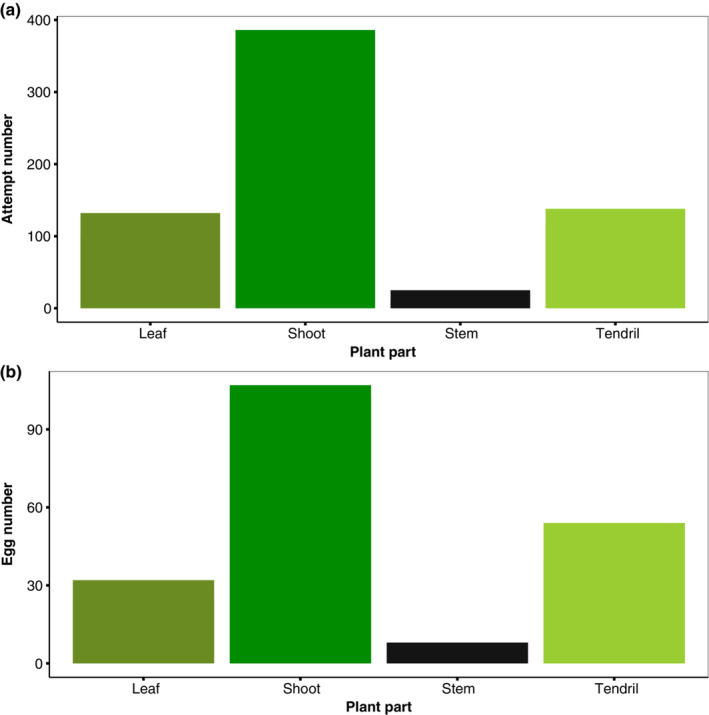
Total number of (a) oviposition attempts and (b) eggs per plant part, throughout the behavioral experiment.

The number of oviposition attempts significantly differed by plant part (LRT = 57.164, df = 3, *p* < .001; Figure 4a). Post hoc tests showed more attempts on shoots compared to leaves (*Z* = 5.268, *p* < .0001), stems (*Z* = 5.988, *p* < .0001), and tendrils (*Z* = 5.506, *p* < .0001). The number of eggs significantly differed by plant part (LRT = 24.704, df = 3, *p* < .001; Figure 4b), but this was not influenced by the UV treatments (the *treatment: plant–part* interaction was non‐significant; Χ^2^ = 0.7731, *p* = .85588). As with the number of eggs, post hoc analyses revealed that more eggs were laid on the shoots compared to leaves (*Z* = 4.85, *p* < .0001), stems (*Z* = 2.780, *p* = .03) and tendrils (*Z* = 2.654, *p* = .04).

### 3.3 Hostplant does not reflect UV and visual models show little‐to‐no stimulation of the UV photoreceptors

**FIGURE 5:**
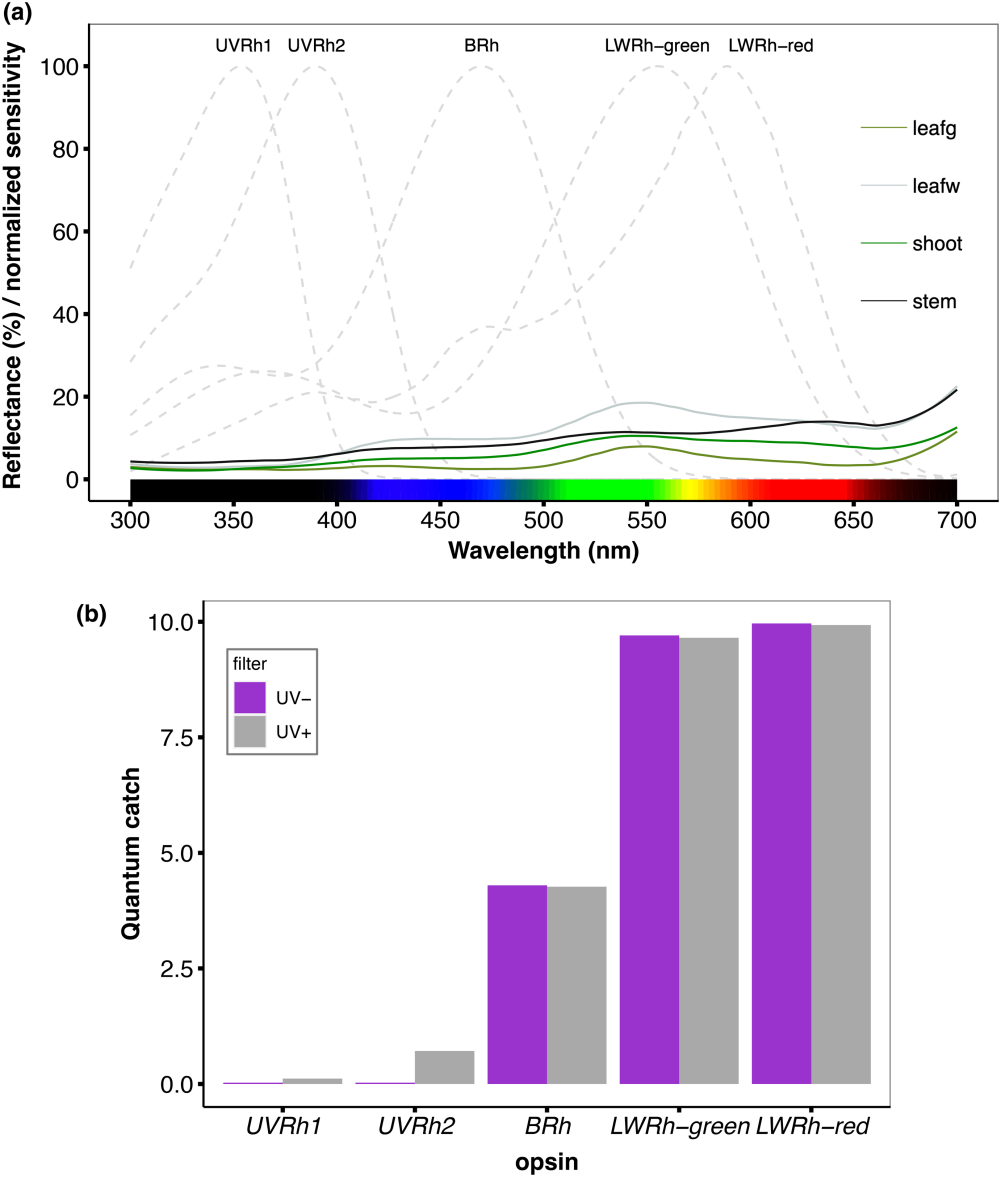
(a) Reflectance spectra of *P. punctata*, leafg represent green parts of the leaf while leafw represent the white spots on the leaves. Gray dotted lines indicate the normalized spectral sensitivities of *H. erato*. (b) Quantum catch estimates of the female *H. erato* visual system when viewing the shoots of *P. punctata* against a green foliage background. Quantum catches were calculated for each opsin *UVRh1*, *UVRh2*, *BRh1*, and *LWRh* including the red “receptor,” which results from red filtering pigments shifting the sensitivity of *LWRh* pigment toward longer wavelengths (McCulloch et al., 2022; Zaccardi et al., 2006). Gray bars show quantum catch estimates under UV+ environment and purple bars show quantum catch estimates under UV− environment. Error bars represent ±standard error.

The spectral reflectance curves of different parts (shoot, stem, leaf, and white patches on the leaf) of the hostplant *P. punctata* are presented in Figure 5a. The observed reflectance curves are characterized by presence of light‐absorbing chlorophyll (Chappelle et al., 1992); reflectance peaks are present at ~550 and >680 nm, and there is low reflectance below 500 nm, with very little reflectance in the UV range (300–400 nm).

To estimate visual perception of the hostplant by females in the UV‐manipulated treatments, we calculated the photoreceptor quantum catches for the shoots of the hostplant—the part where most eggs were laid—against a green foliage background under each experimental condition (UV+/UV−; Figure 5b). Under natural sunlight (UV+), our models predicted minimal stimulation of *UVRh2* and *UVRh1* was not stimulated. In UV‐absent conditions (UV−), neither *UVRh1* nor *UVRh2* were stimulated. In contrast, the blue photoreceptor (*BRh*) and the long‐wavelength photoreceptors (*LWRh‐green and LWRh‐red*) were similarly stimulated under both lighting conditions. The long‐wavelength receptor with red filtering pigments (*LWRh‐red*) had the highest quantum catch, followed closely by the long‐wavelength opsin (*LWRh‐green*; Figure 4b).

## 4 DISCUSSION

Vision plays a crucial role in *Heliconius* behaviors, including mate choice (Estrada & Jiggins, 2008; Merrill et al., 2014), foraging (Toure et al., 2020), and hostplant selection (Gilbert, 1982). Through a gene duplication event at the base of the *Heliconius* genus, these butterflies gained a secondary UV‐sensitive opsin (Briscoe et al., 2010; Bybee et al., 2012). However, levels of expression of this opsin varies both between and within species. For example, numerous species have independently lost expression of one of the two UV opsins, with documented pseudogenization events (McCulloch et al., 2017). In *H. erato*, UV opsin expression is sexually dimorphic: females express both UV opsins, whereas males only express one (McCulloch et al., 2016).

In a recent laboratory study, male and female *H. erato* differed in UV wavelength discrimination (Finkbeiner & Briscoe, 2021). However, the ecological pressures that have driven these sex‐specific differences in visual perception remain unresolved. Given the differences in life history between male and female *Heliconius* butterflies, we predicted that UV wavelength discrimination might benefit female‐specific behaviors such as oviposition. A strong body of evidence suggests the importance of visual cues for finding suitable hostplants for oviposition in *Heliconius* females (De Nardin & De Araújo, 2011; Dell'Aglio et al., 2016; Gilbert, 1982; Williams & Gilbert, 1981). However, our experiments suggest that UV wavelength discrimination in *H. erato* females is not an adaptation associated with oviposition behaviors. In addition to visual information *Heliconius* and other butterflies use other modalities during host choice, including chemosensation (Briscoe et al., 2013) and tactile information (Thiele et al., 2016). It is possible that at shorter distances, these other sensory modalities may play a primary role in hostplant decision.

In our experiments, the availability of UV light did not influence the number of oviposition attempts, nor the number of eggs laid by *Heliconius* females in the two studied species. While it is plausible that the lack of statistical power may have contributed to the non‐significant findings regarding the effect of UV presence on the number of eggs, it is unlikely to be the sole explanation. Our spectral reflectance measures of the hostplant *P. punctata* provides a more likely explanation for these results. Overall, we found only minimal UV reflection in any of the hostplant parts of *P. puctata* where female butterflies laid eggs (Figure 4a). These results are consistent with the fact that UV reflectance is usually—but not always—low on leaves (Archetti et al., 2009), whereas, in contrast, many flowers reflect UV (Arnold et al., 2008). We also used female *H. erato*‐specific opsin sensitivities (McCulloch et al., 2016, 2022) to estimate the photoreceptor quantum catches when viewing the shoots of *P. punctata*—where most eggs were laid—in both light conditions (UV+ & UV−). Neither UV photoreceptor (*UVRh1* or *UVRh2*) was stimulated under the UV‐absent conditions, and under natural sunlight (UV‐present), only *UVRh2* was minimally stimulated (Figure 4b). The absence of UV reflectance in the hostplant and little‐to‐no stimulation of the UV photoreceptors suggests that UV discrimination does not directly affect *Heliconius* female oviposition. However, it is important to note that these conclusions are based on estimates of visual system stimulation which are inherently limited (Dell'Aglio et al., 2018; Drewniak et al., 2020; Finkbeiner & Briscoe, 2021), further highlighting the importance of our behavioral studies.

The circuitry required for UV discrimination is metabolically costly and may have trade‐offs with other components of color vision (McCulloch et al., 2016). Our experiments suggest that UV perception in *H. erato* females is not used during oviposition. An alternative is that *H. erato* females may use UV discrimination to detect previously laid eggs. Because of cannibalism in *Heliconius* larvae, females avoid ovipositing in the presence of conspecific eggs on the hostplants. However, neither *H. erato* eggs nor *Passiflora* egg‐mimics reflect wavelengths in the UV range (300–400 nm; Finkbeiner & Briscoe, 2021) so this explanation seems unlikely.

Another possibility is that UV wavelength discrimination is used in female mate choice. In other butterfly groups, such as *Colias* and *Eurema*, UV reflectance is used by females for conspecific recognition and mate choice (Kemp, 2008; Silberglied & Taylor, 1973). In *Heliconius*, UV opsin duplication co‐occurred with the evolution of a yellow pigment (3‐hydroxyDL‐kynurenine) that reflects UV (Briscoe et al., 2010; Bybee et al., 2012) and additionally in some species of *Heliconius* such as *H. doris*, the structural colored scales reflect UV (Wilts et al., 2017). However, there are populations of *H. erato* which do not show these yellow patterns, and it is currently unknown whether variation in UV vision exists between populations. Experiments have shown that both male and female *H. erato* individuals prefer to approach UV+ over UV− models (Finkbeiner et al., 2017); however, *Heliconius* females do not generally approach males to solicit mating, and these experiments cannot distinguish between UV‐guided mating preference behaviors, or more general attraction to UV reflecting cues, which are common in flowers used by these butterflies (see below). Other experiments have manipulated UV reflectance on the wings of *H. erato* and its co‐mimic *H. melpomene*, by applying UV‐blocking sunscreen, and have found that *H. erato* males more often approached *H. melpomene* females when the UV signal was blocked (Dell'Aglio et al., 2018). However, this does not explain the sexual dimorphism in UV opsin expression in these species. Nevertheless, visual modeling does suggest that female *H. erato* may be able to distinguish between the yellow colors of *H. erato* and *H. melpomene* (Dell'Aglio et al., 2018), so although there is little evidence that wing colors play a role in female mate choice in *Heliconius*, it remains an intriguing hypothesis.

A more likely alternative function of UV discrimination in *H. erato* females could relate to foraging. Most insects that forage on flowers, such as bees and butterflies, can perceive UV (Briscoe & Chittka, 2001). Analysis of the reflectance of *Psychotria* and *Psiguria*—two pollen plants used by *H. erato*—found a UV component on the reflectance spectrum of their flowers (Finkbeiner & Briscoe, 2021). Due to egg production, female *Heliconius* have higher nutrient requirements than males, and may need to invest more in foraging for pollen resources. In particular, in *H. charathonia*, which also has sexually dimorphic vision (McCulloch et al., 2017), females have been shown to collect significantly more pollen than males (Mendoza‐Cuenca & Macías‐Ordóñez, 2005). Using a similar experimental design as the one used in the present study, future research could investigate the function of UV discrimination in the context of foraging.

An important caveat of our study is that we used individuals from populations collected from the wild on the western slopes of the Andes in southern Ecuador. Previous studies, which reveal evidence of sexually dimorphic expression of the UV‐opsins, used *H. erato petiverana* individuals from Costa Rica (McCulloch et al., 2016, 2017). The same subspecies, *H. e. petiverana*, and supplier was also used for laboratory‐based UV wavelength discrimination experiments (Finkbeiner & Briscoe, 2021). The most recent common ancestor of the *H. erato* clade dates to 200,000–500,000 years ago and since then, over 15 *H. erato* populations with different wing patterns have evolved (Van Belleghem et al., 2017). Gene expression evolution can occur rapidly, especially in visual systems (Seehausen et al., 1997; Nandamuri et al., 2017). Therefore, *H. erato* populations might differ in their opsin expression patterns, though this has not yet been explored.

Experimental manipulation of ambient light using filters is a common method to simulate natural light environments or remove specific wavelengths altogether. Studies in a range of taxa, including aquatic (Hiermes et al., 2021; Wright et al., 2017) and terrestrial organisms (Greenwood et al., 2002; Honkavaara et al., 2008), have used this technique to investigate the evolution of animal visual systems and associated behaviors. However, to our knowledge, this is one of the very few studies (Veen et al., 2017) that used filters to modify natural sunlight conditions in a behavioral experiment. Using natural sunlight conditions as opposed to standardized artificial lighting is likely to better represent the lighting conditions found in these species habitats and may elicit more natural behavior. However, experiments under natural sunlight conditions are subject to considerable light intensity variation (see Figure S3). Thus, an unintentional difference in light intensity may affect the results. Indeed, weather conditions significantly affected oviposition attempts and the number of eggs laid in our study (Figure S4). Butterflies made more attempts and laid more eggs on sunny days than on overcast weather. For this reason, the majority of behavioral studies that have manipulated the UV presence using UV‐blocking filters have used standardized artificial lighting conditions (Greenwood et al., 2002; Hiermes et al., 2021; Honkavaara et al., 2008; Lewis et al., 2000). Nevertheless, under natural conditions—particularly in rainforests—light intensity varies rapidly (Endler, 1993), which will be better reflected by experiments manipulating wavelength under more natural conditions such as ours.

In our study, weather significantly affected oviposition attempts and the number of eggs laid. Compared with overcast weather, butterflies made more attempts and laid more eggs on sunny days (Figure 4). This is in line with other studies of *Heliconius* butterflies where weather and light conditions have been documented to affect preference behaviors (Hausmann et al., 2021), and butterflies are more active on sunny days (Jiggins, 2017; Mérot et al., 2015). Similarly, weather and light condition influence the behavior in other Lepidoptera taxa such as the activity in Noctulid moths (Yela & Holyoak, 1997), habitat use, and distribution in satyrine butterfies (Ide, 2002) and mating activity in the *Precis coenia (*Mcdonald & Nijhout, 2000). Overall, these findings highlight the broader significance of weather and light as key factors shaping the behavior of butterflies and moths across different taxa.

In conclusion, *Heliconius* color vision is fundamental for guiding behaviors, including mate choice, oviposition, and foraging. In contrast to *H. erato* males, *H. erato* females express two UV‐sensitive opsins and can discriminate between UV wavelengths, but the selective pressures driving sexual dimorphism remain unresolved. By manipulating the light environment under naturalistic conditions, we show that UV perception in *H. erato* females is unlikely to be an adaption relating to oviposition behaviors. However, it is important to note that our findings are based on relatively few specimens due to limitations in our stocks. To strengthen the validity of our results, future experiments should consider expanding the sample size. Thus, the selective pressures driving sexual dimorphism remain unresolved. Further research is required to better understand the evolutionary processes that have sex‐specific differences in visual perception in *Heliconius*.

## AUTHOR CONTRIBUTIONS

**Jose Borrero:** Conceptualization (supporting); data curation (lead); formal analysis (lead); investigation (lead); methodology (equal); visualization (lead); writing – original draft (lead). **Daniel Shane Wright:** Conceptualization (equal); investigation (supporting); methodology (equal); supervision (equal); writing – review and editing (equal). **Caroline Nicole Bacquet:** Resources (supporting). **Richard M. Merrill:** Conceptualization (equal); formal analysis (supporting); funding acquisition (lead); project administration (lead); resources (lead); supervision (equal); writing – review and editing (equal).

## FUNDING INFORMATION

This research was funded by a European Research Council (ERC) Starter Grant (851040) to R.M.M.

## CONFLICT OF INTEREST STATEMENT

The authors have no conflict of interest to declare.

### OPEN RESEARCH BADGES

This article has earned an Open Data badge for making publicly available the digitally‐shareable data necessary to reproduce the reported results. The data is available at https://doi.org/10.5061/dryad.7m0cfxq0h.

## Supporting information


Figures S1–S6

## Data Availability

The data supporting this study are available in the Dryad repository (https://datadryad.org) under the accession number https://doi.org/10.5061/dryad.7m0cfxq0h.
