# Supplementary material for: Oviposition behavior is not affected by ultraviolet light in a butterfly with sexually‐dimorphic expression of a UV‐sensitive opsin
Source: Ecol Evol. 2023 Jul 4;13(7):e10243. doi: 10.1002/ece3.10243 (PMC10318619; doi:10.1002/ece3.10243)
Supplement: Supplementary file 1 — Figures S1–S6 [file ECE3-13-e10243-s001.docx]

Supplementary Figure 1: Normalized spectral sensitivities of *Heliconius erato*. (B) Males possess opsins with peak sensitivities: UV 390 nm (*UVRh2*), blue 470 nm (*BRh*) and long-wavelength (green) 555 nm (*LWRh*) (A) Females possess an additional UV opsin (*UVRh1*) that has a peak sensitivity at 355 nm. Additionally, *H. erato* possesses a fifth receptor class, with a peak at ∼590 nm due to filtering of the green rhodopsin by a red filtering pigment (red dashed line, from McCulloch *et al*. 2022).

Supplementary Figure 2: Experimental cage set up. (A) Schematic view of the cage used for the behavioural experiment. Cage on the top left shows experimental cage under UV- absent conditions when fitted with a UV-blocking filter. Cage on the top right shows experimental cage under UV+ present conditions, when fitted with a clear filter. Filters were placed on the east and south-ward facing sides of the cage which received most of the incoming natural sunlight. (B) Picture of the experimental cage fitted with UV-blocking filter with a *Passiflora punctata* hostplant.

Supplementary Figure 3: Experimental light irradiance in the UV-Vis range under different weather conditions. Purple line represents irradiance in cage with UV-blocking filter (UV-), grey line represents irradiance in the cage with clear filter (UV+). Shaded areas represent ± one standard error. Left column shows the irradiance spectra under overcast conditions (100% cloud coverage), middle column shows the irradiance spectra under cloudy conditions (>50% c.c.) and right column shows the irradiance under sunny conditions (<50 % c.c.). Top three rows show sides of the cage fitted with filters (1) downwelling, (2) side-welling out (southeast facing) (3) side welling left (east facing). Bottom two rows show sides of the cages that were not fitted with filters (4) side welling in (north facing) & side welling right (northwest facing).

Supplementary Figure 4: Light irradiance in the UV range (<400 nm) in the experimental cages in different weather conditions. Violet line represents irradiance in cage with UV-blocking filter (UV-), grey line represents irradiance in the cage with clear filter (UV+). Shaded areas represent ± one standard error.

Supplementary Figure 5: Average time to first (A) oviposition attempt (B) egg laid. Grey bars represent number of attempts/eggs in the control treatment (UV+) and purple boxes represent the number of attempts/eggs in the UV- light environment.

Supplementary Figure 6: Egg count per treatment by individual. Females marked with C represent *H. erato cyrbia* while *H. himera* individuals are represented with an H.
